# Prospective validation of mean metacarpophalangeal joint extension as a measure of diabetes-related fibrotic hand manifestations

**DOI:** 10.3389/fcdhc.2025.1650796

**Published:** 2026-01-15

**Authors:** Sanat Phatak, Sarita Jadhav, Rucha Wagh, Parth Ladha, Rishi Nalkande, Rutvij Tope, Harsh Balbudhe, Rohan Shah, Smita Dhadge, Pranay Goel, Jennifer L. Ingram, Chittaranjan Yajnik

**Affiliations:** 1 King Edward Memorial Hospital and Research Centre, Pune, India; 2 Byramjee Jeejeebhoy Government Medical College, Pune, India; 3 Indian Institute of Science Education and Research (IISER), Pune, India; 4 Division of Pulmonary, Allergy and Critical Care, Department of Medicine, Duke University Medical Centre, Durham, NC, United States

**Keywords:** clinical instrument, COSMIN, diabetic cheirarthropathy, fibrosis, hand

## Abstract

**Introduction:**

Hand conditions in diabetes, namely, limited joint mobility (LJM), flexor tenosynovitis (FT), carpal tunnel syndrome (CTS), and Dupuytren disease (DD), share a common pathophysiological process involving pro-fibrotic inflammation in flexor structures. A unified, quantitative measure of disease severity across these conditions is lacking, limiting correlational research. We evaluated mean metacarpophalangeal (MCP) joint extension as a potential measure of severity.

**Methods:**

We assessed 2,405 adults, including individuals with type 1 diabetes (n=291), type 2 diabetes (n=877), prediabetes (n=326), and non-diabetic controls (n=911). MCP extension was calculated as the average maximum passive extension of the second to fifth fingers, measured with a protractor. Validity was determined by correlating MCP extension with physician-rated severity (convergent) and hand grip strength and the Duruöz Hand Index (DHI, both divergent). Inter-rater reliability was tested in 128 individuals, and sensitivity to change was evaluated in 143 participants assessed at two time points.

**Results:**

Mean MCP extension was significantly lower in individuals with all hand conditions (42.4° LJM, 42.8°FT, 39.9° DD, 51.7 °CTS) than in those without (58.6°, all p<0.05). MCP extension correlated with physician-rated severity (−0.5, p<0.01) and weakly with DHI (R^2^ = 0.03) and grip strength (R^2^ = 0.07). Inter-rater reliability was strong (ICC 0.72), and MCP extension demonstrated sensitivity to change, worsening over 8 months (SRM −0.61).

**Conclusion:**

Mean MCP extension is a valid, reliable, and responsive measure for assessing fibro-inflammatory hand conditions in diabetes.

**Clinical Trial Registration:**

https://ctri.nic.in/Clinicaltrials/login.php, identifier CTRI/2020/12/030057.

## Introduction

The observation that some patients with diabetes have limited joint mobility in the hand was first made by Rosenbloom et al. in 1974 (1). The spectrum of hand manifestations that occur within diabetes, termed “diabetic cheiroarthropathy” or “diabetic hand,” now includes limited joint mobility (LJM), flexor tenosynovitis (FT) including stenosing FT or trigger finger, Dupuytren’s contracture (DC), and carpal tunnel syndrome (CTS) (2).

The clinical correlates of these hand syndromes with other organ manifestations within diabetes are unclear. While some reports show an increased prevalence of hand disorders in patients with microvascular complications of diabetes mellitus (3), others do not (4). Despite their heterogeneity, at least three of these manifestations (LJM, FT, and DC) share the common pathophysiologic process of profibrotic inflammation, demonstrated on histopathology (5). CTS may be secondary to fibrotic thickening of the other structures in the carpal tunnel but may also be partly microvascular (6). We previously hypothesized that these easily accessible manifestations may reflect a global profibrotic trajectory in internal organs and could potentially serve as a clinical biomarker (7).

One hurdle in establishing such correlations is the lack of a clinical tool that can serve as a proxy measure of the amount of inflammation-fibrosis in the hand, unifying all these manifestations. The Duruöz Hand Index (DHI) is a functional score that has been validated for use in diabetes (8). However, barring early FT and CTS, these conditions are rarely painful and are functionally limiting only when they produce severe mobility restriction (9). In this situation, a clinical metric that banks on the biology of fibrosis (resistance to stretch) rather than on perceived clinical and functional effects (functional limitation, pain) is likely to be more useful. In LJM, FT, and DC, profibrotic inflammation occurs in one or more of the structures on the flexor aspect of the palm. In severe cases of LJM, contractures ensue, leading to the classical “prayer sign” (10). However, finger extension is likely to be limited much earlier than this state.

We hypothesized that such limitation would reflect in a reduced angle of maximum passive extension at the metacarpophalangeal (MCP) joint, when the hand is approximated on a flat surface. In a type 1 diabetes cohort, we showed that the mean of the angles of passive extension at the MCP joint of the second to fifth fingers (henceforth, mean MCP extension) was reduced in those with hand manifestations (9). Mean MCP extension is easy to calculate at the community level and does not need expensive equipment nor extensive training. Furthermore, this metric correlated with the presence of fibrotic tissue thickening on MRI in the type 1 diabetes patients studied.

In this study, we evaluate the performance of MCP extension as a measure of hand manifestations in an expanded population that includes type 2 diabetes, prediabetes, and non-diabetic controls in addition to type 1 diabetes. We use COnsensus-based Standards for the selection of health Measurement Instruments (COSMIN) methodology to describe validity, reliability, and responsiveness, using separate cohorts for different aims, which add credibility to its potential use as a clinical metric of fibro-inflammatory flexor hand involvement in these conditions (11).

## Methods

### Patients

We studied adults (>18 years) with type 1 diabetes, type 2 diabetes, prediabetes, and healthy controls (main cohort). All individuals were seen in the study period between April 2021 and April 2024 in the diabetes, internal medicine, and rheumatology clinics at the King Edward Memorial Hospital, a tertiary care hospital in Pune, India. Type 2 diabetes and prediabetes were diagnosed using American Diabetes Association criteria cutoffs (12). Type 1 diabetes was diagnosed clinically, using criteria of diagnosis prior to 30 years, documentation of ketosis, and insulin dependence. Those with prediabetes and healthy controls were invited from relatives or friends accompanying patients, as well as from within the Pune Maternal Nutrition Study (PMNS) longitudinal cohort (13). Independent recruitments were performed for inter-rater reliability and sensitivity-to change studies, and these are described under those headings (reliability cohort and responsiveness cohort, respectively).

**Figure 1:**
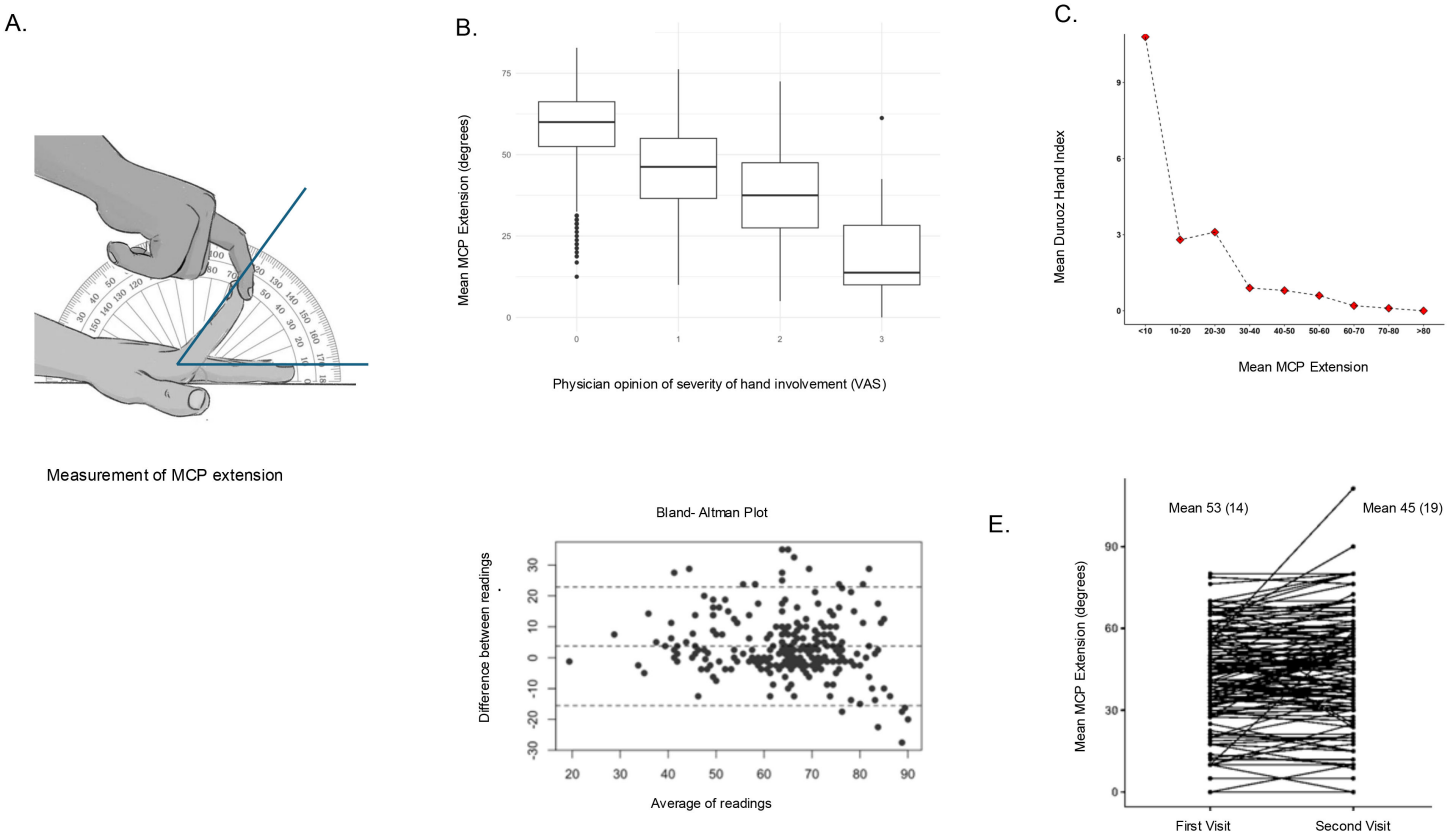
Behavior of mean metacarpophalangeal (MCP) joint extension in diabetes-related flexor fibroinflammatory hand manifestations (diabetic hand syndromes). **(A)** Clinical methodology of measuring metacarpophalangeal joint extension in the second finger; mean MCP extension is an average of the angle in the second to fifth digits. The patient was sat on a chair and the hand kept on a desk with the palm approximated. Passive stretch was applied to each finger in sequence. **(B)** Convergent validity of mean MCP extension: correlation with physician’s opinion of severity of hand manifestations. **(C)** Divergent validity of MCP extension: correlation with the Duruöz Hand Index, a validated measure of hand function. **(D)** Bland–Altman plot showing inter-rater reliability between two raters. **(E)** Change in mean MCP extension in patients with hand manifestations over an average duration of 8 months, receiving standard treatment.

We collected demographic and clinical information and data on diabetes duration, medications, and micro- and macrovascular complications from patients’ files. Comprehensive clinical evaluations included a standardized examination for signs (Tinel sign, tenosynovial thickening, triggering, skin thickening, prayer sign). We measured hand grip strength using a Jamar dynamometer (Patterson Medical, Warrenville, IL). Grip strength was assessed following standardized protocol recommendations, with the participant seated, elbow flexed at 90°, and forearm in a neutral position. The mean of three consecutive measurements from the dominant hand was recorded. Patients were then asked to complete the DHI, a validated questionnaire that assesses functional disability of the hand in daily living activities, to capture patient-perceived limitations, particularly in tasks requiring dexterity or grip (8). Mean MCP extension was calculated as an average of maximum passive extension at the second, third, fourth, and fifth fingers of each hand. (**Figure 1A**) The palm was approximated on a flat horizontal surface, and the fingers were extended upward. A protractor was used to measure the angle between the finger skin and the flat surface. The patient was in the sitting position with elbow flexed. All measurements were performed by trained research staff (SJ, RW, RS, RT, HB).

A rheumatologist (SP) or a diabetes specialist (SD) independently evaluated the presence and type of hand manifestations based on clinical examination findings (e.g., triggering, nodules, skin thickening, pain, and prayer sign) and a structured form capturing signs and symptoms. Because MCP extension can be altered by several non-diabetic conditions, we excluded individuals with inflammatory arthritis such as rheumatoid arthritis, prior significant hand trauma or surgery, neurological conditions causing contractures, and palmar dermatologic diseases such as psoriasis. We assessed inter-rater reliability between the two physicians on 30 randomly selected patients and found an agreement of 0.89 (Cohen’s kappa). These ratings were used for diagnostic classification and not as the primary severity scale.

LJM was diagnosed when there was skin thickening leading to flexion contractures, or a prayer sign. Those with thickened palmar skin alone but without a prayer sign were labelled as skin thickening (ST). FT was diagnosed when there was flexor tendon pain or tenderness, beading, triggering, and/or palpable tendon crepitus. DD was diagnosed when palmar fascia thickening was palpable as a nodule or had resulted in a contracture. CTS was diagnosed when there were neuropathic symptoms or signs in the median nerve distribution or the Tinel/Phalen sign was positive. For convergent validity, a separate physician-rated visual analog scale (0–3) for severity of hand involvement was used.

### Validity studies

Construct: The hand manifestations of diabetes can be considered a medical construct that results in stiffness and resistance to stretch within the flexor compartment of the hand. This condition arises from an initial period of inflammation (especially seen in subacute flexor tenosynovitis, leading to palmar pain) and, later, excessive collagen deposition, leading to restricted movement. The construct focuses specifically on quantifying the mechanical properties of the affected tissues, namely, their resistance to stretching forces, independent of pain perception or functional loss.

Convergent validity: We examined correlations between mean MCP extension and the physician’s subjective opinion for severity of fibro-inflammatory hand manifestations, using Spearman’s rank correlation test.

Divergent validity: We examined correlations of MCP extension with hand grip strength (measuring flexor muscle strength and nerve function) and DHI (measuring hand pain and functional loss), using Spearman’s rank correlation test. We selected DHI and grip strength to assess divergent validity because they represent distinct constructs: DHI captures patient-perceived hand function and pain, whereas grip strength reflects neuromuscular performance. Since mean MCP extension is intended to reflect passive mechanical stiffness due to fibrosis rather than active function or pain, we expected only weak correlations with these measures.

### Reliability studies

Inter-rater reliability: Inter-rater reliability was assessed on an independent set of patients (reliability cohort), a mixture of patients with diabetes, patients with rheumatic disease, and healthy controls seen at the rheumatology and internal medicine outpatient clinics. Two examiners (PL and RN) received a brief training on measuring MCP extension. Both measured and calculated mean MCP extension for each hand, without access to the other’s measurement. Data regarding diagnosis, age, and hand pain were extracted from files. We calculated single-score intra-class correlation (two-way model, type agreement) and performed Bland–Altman analysis for agreement between the two raters. Cohen’s kappa was calculated to see if both raters could pick up those with restricted mean MCP extension, taken arbitrarily as less than 40°.

### Responsiveness to change

From within the main cohort, we studied individuals with at least one hand manifestation who had been examined twice, with an interval of at least 6 months. Patients received standard care during this period, which could include physical therapy, analgesics, or local corticosteroid injections, based on clinical indication. No uniform treatment protocol was mandated. Mean MCP extension and DHI were measured at both visits. Responsiveness to change was calculated as a standardized response mean (SRM) using change scores between two time points. Effect size was denoted with a Cohen’s D.

Statistical analyses: Data are presented as frequencies, mean, and standard deviation as appropriate. Comparison in mean MCP extension between groups (types of diabetes vs. controls, those with hand manifestations versus those without) was done using analysis of variance (ANOVA), adjusted for age and sex. We performed a multivariable linear regression in the diabetic subpopulation to explore associations between mean MCP extension and clinical variables including age, sex, diabetes duration, insulin use, BMI, microvascular and macrovascular complications, liver disease, and adhesive capsulitis. Individual statistical analyses for each goal are outlined in the respective section. All analyses were done using R. (R Core team, 2024- R Foundation for Statistical Computing, Vienna, Austria.).

**Ethics:** Ethics permission was granted by the KEM Hospital Research Centre Ethics Committee (KEMHRC/RVC/EC/1518). All patients signed informed consent forms. This study is registered with the Clinical Trials Registry of India (CTRI/2020/12/030057, dated 28/12/2020). All data are stored at the Diabetes Unit, KEM Hospital Research Center. All patients who were found to have hand manifestations were offered management including local glucocorticoid infiltration for trigger fingers, physical therapy, and referrals to specialists (such as neurology or hand surgery, as indicated).

### Role of funding

This study is funded through a DBT/Wellcome India Alliance Clinical and Public health fellowship (IA/CPHE/19/504607, awarded to SP). The funding body had no role in study design or analysis.

## Results

### Patients’ characteristics (main cohort)

We studied 2,405 participants with type 1 diabetes (n=291), type 2 diabetes (n=877), and prediabetes (n=326) and non-diabetic controls (n=911). Patients with type 1 diabetes had a mean age of 28.2 years, mean duration of diabetes of 14.8 (SD 9.9) years, and mean HbA1c of 9.3 ± 2.4 (available in 332). Patients with type 2 diabetes (53% female) had a mean age of 56.4 ± 11.8 years, mean duration of diabetes of 12.1 = −8.8 years, and mean HbA1c of 8.6 ± 2.0 (available in 848). Controls (47% female) had a mean age of 40.1 ± 16 years. Approximately one-third of patients with diabetes and prediabetes had at least one hand manifestation present. (**Table 1**) LJM and ST were the most common, whereas isolated CTS was the rarest across patient groups. Individuals with type 1 diabetes were most likely to have multiple manifestations. Non-diabetic controls had hand manifestations, most commonly LJM and skin thickening, in 24%. Hand manifestations were more common in both type 1 diabetes, type 2 diabetes (a 10% difference), and prediabetes as compared with non-diabetic controls. (all p< 0.01).

**Table 1:** Prevalence of flexor fibro-inflammatory hand manifestations across patient groups.

Group	Type 1 diabetes	Type 2 diabetes	Prediabetes	Normal glucose tolerance
Number	291	877	326	911
Female (%)	170 (58.6)	463 (52.8)	159 (48.8)	423 (46.4)
Mean age, years (SD)	29.1 (10.5)	57.2 (11.7)	44.0 (12.2)	38.4 (12.2)
Any hand manifestation N, %	98 (34)	317 (36)	102 (31)	222 (24)
LJM N (%)	36 (36.7)	125 (39.8)	54 (52.9)	91 (41.9)
ST N (%)	22 (22.4)	89 (28.3)	41 (40.2)	84 (38.7)
FT N(%)	32 (32.7)	83 (26.4)	4 (3.9)	36 (16.6)
DD N(%)	6 (6.1)	11 (3.5)	1 (1.0)	3 (1.4)
CTS N(%)	2 (2.0)	6 (1.9)	2 (2.0)	3 (1.4)
More than one manifestation N (%)	15 (15.3)	21 (6.62)	2 (1.9)	6 (2.7)

SD, standard deviation; LJM, limited joint mobility; ST, skin thickening—not classifying as LJM; FT, flexor tenosynovitis; DD, Dupuytren’s disease; CTS, carpal tunnel syndrome.

MCP extension in individual manifestations and clinical associations: Mean MCP extension was lower in type 1 diabetes (mean 53.4°, p= 0.01), type 2 diabetes (mean 50.3°, p < 0.01), and prediabetes (mean 54.5°, p=0.05) than controls (mean 57.1°). Pooling all patient groups, those with no hand manifestations (n= 1666) had a mean MCP extension of 58.5°. (**Table 2**) Mean MCP extension was lower by approximately 20° in those with LJM, ST, FT, and DD (all p<0.01) and to a lesser extent in CTS. A gender-adjusted regression model in the diabetic population showed that the mean MCP angle was associated with diabetes duration (p<0.01) and microvascular complications (p=0.03) but not with insulin use, body mass index, macrovascular complications, and history of adhesive capsulitis or liver disease.

**Table 2:** Mean metacarpophalangeal (MCP) joint extension and measures of hand function in patients with individual hand manifestations.

Outcome measure	None	LJM	p-value	ST	p-value	FT	p-value	DD	p-value	CTS	p-value
MCP extension (degrees) (mean, SD)	58.57 (11.01)	42.48 (15.5)	<0.01	43.36 (12.6)	<0.01	42.88 (14.7)	<0.01	39.94 (18.4)	<0.01	51.73 (13.7)	0.05
DHI (mean, SD)	0.18 (1.52)	0.71 (4.85)	<0.01	0.37 (2.14)	0.08	7.27 (13.49)	<0.01	0.42 (1.96)	0.8198	7.23 (11.13)	<0.01
Grip strength (mm Hg) (mean, SD)	49.32 (19.59)	45.13 (19.42)	< 0.01	49.83 (19.81)	0.61	33.12 (14.9)	<0.01	54.32 (17.94)	0.1057	38.26 (18.51)	0.04

SD, standard deviation; LJM, limited joint mobility; ST, skin thickening—not classifying as LJM; FT, flexor tenosynovitis; DD, Dupuytren’s disease; CTS, carpal tunnel syndrome; DHI, Duruöz Hand Index.

### Convergent validity

The mean MCP extension angle correlated with physician-rated severity of fibro-inflammatory hand involvement, assessed using a 0–3 visual analog scale (0 = no involvement, 3 = severe involvement) (Spearman correlation coefficient −0.50, p<0.01, **Figure 1B**).

### Divergent validity

DHI was elevated in LJM, FT, and CTS but not in ST and DD. The MCP extension angle correlated weakly with DHI (R^2^ = 0.03, p<0.01), and DHI was elevated mainly in those with MCP extension below 30°. (**Figure 1C**) Grip strength was statistically lower in LJM, FT, and CTS, and correlated weakly with MCP extension. (R^2^ = 0.07, p<0.01).

### Inter-rater reliability

The reliability cohort (n=128) included 32 patients with inflammatory or degenerative arthritis, 22 with diabetes, and 2 with hypothyroidism; the rest were healthy controls. There were 28 who had hand pain, ranging on a pain visual analogue scale from 1 to 6. There were 256 pairs of observations for MCP extension. Two raters achieved a single-score intra-class correlation of 0.72 (95% confidence intervals 0.61 to 0.79, p <0.01). On Bland–Altman analysis (**Figure 1D**), the mean difference was 3.39° (SD 9.49). Cohen’s kappa for agreement in picking up those with restricted MCP extension <40° was 0.81 (p <0.01).

### Responsiveness to change

In the main cohort, 143 participants (all with at least one hand manifestation, 55 with type 1 diabetes, 82 with type 2 diabetes, and 6 non-diabetes controls) had two evaluation visits at an average duration of 8 months. Mean MCP extension reduced from 53 (14.6)° to 45 (19.4)° with a standardized response mean of −0.61 (p<0.01) and a Cohen’s D of 0.48. (**Figure 1E**) There was no significant difference in the change between type 1 and type 2 participants. The mean DHI increased from 0.77 to 3.2 (p <0.01) (data not shown).

## Discussion

Diabetes-related inflammatory-fibrotic manifestations of the hands are common. A lack of a unifying and simple methodology to measure these manifestations clinically may have precluded examining whether they are biomarkers of a global multiorgan profibrotic trajectory. We show that mean MCP extension is an easy and quantitative measurement that picks up most of these hand manifestations, even in asymptomatic stages. Using COSMIN methodology, we show that it is a valid and reliable metric on a large, heterogenous prospective cohort with varying manifestations. We lay grounds for its use in both assessing and describing the severity of hand problems themselves, as well as establishing correlations with internal organ fibrosis or serological biomarkers of ongoing profibrotic processes.

In our study, individuals with diabetes-related hand manifestations exhibited a 20° reduction in mean MCP angle as compared with those without, a difference we believe is clinically significant and possible to ascertain to the eye. Although grip strength also differed statistically between the groups (45 vs. 49 mm Hg in LJM versus normal), it would not be considered clinically significant. MCP extension was also reduced in LJM, skin thickening, and DD, which are all painless conditions. This finding contrasted with DHI, which was not significantly higher in these three conditions and thus could pick up only CTS and FT, which affect hand function due to neuropathy or pain respectively. We did find a low prevalence of CTS in our cohort (≈2%), which contrasts with higher rates reported in diabetes cohorts evaluated using nerve conduction studies. In our study, CTS was identified solely through clinical symptoms and signs in an unselected outpatient population. These methodological differences likely account for the lower observed prevalence and represent an acknowledged limitation of our dataset.

**Figure 2:**
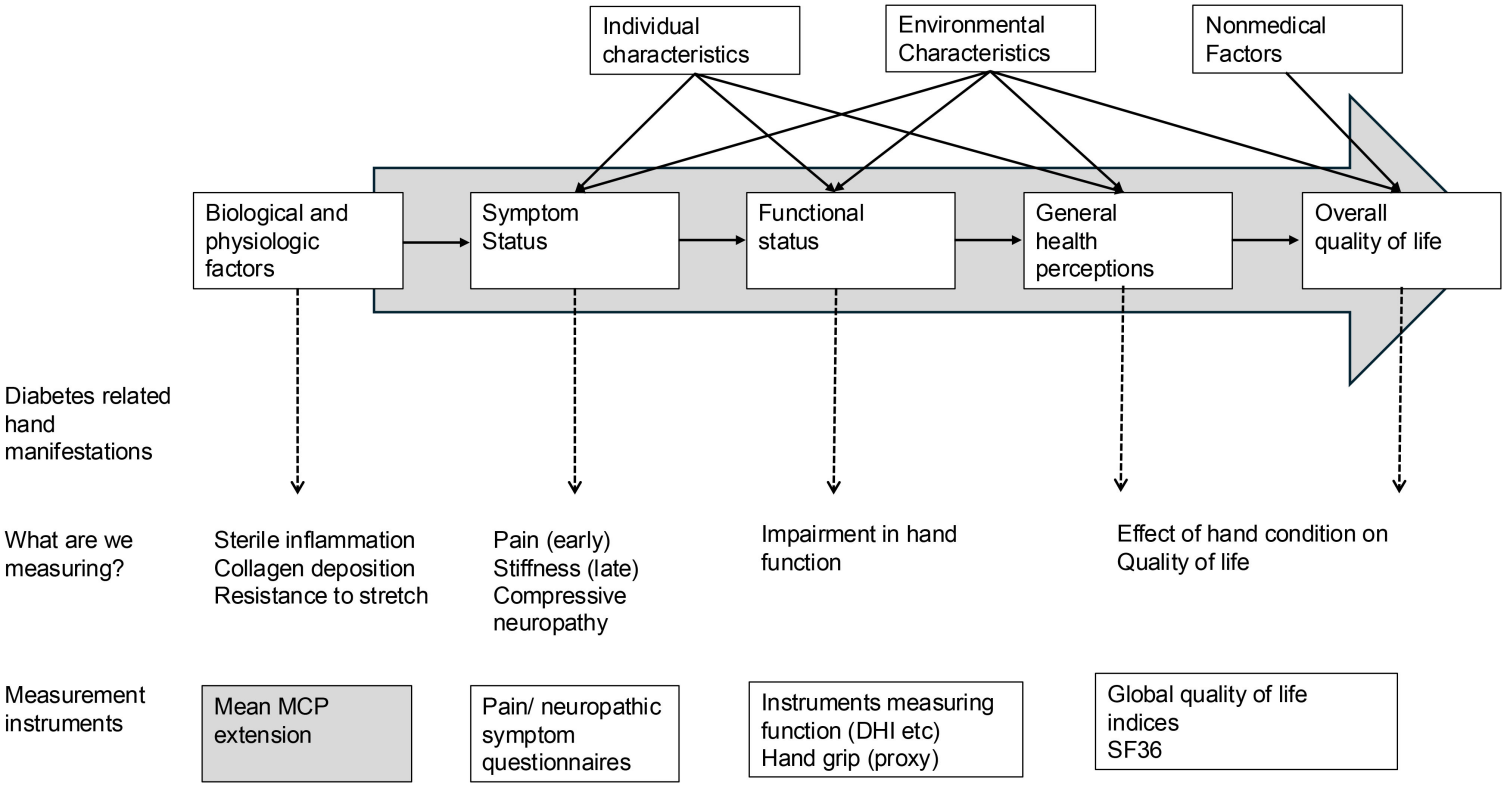
Wilson and Cleary framework (1995) to link biological factors to health-related quality of life, as applied to diabetes-related hand manifestations.

The Wilson and Cleary conceptual model (1995) links biological and physiological variables to health outcomes through a continuum that includes symptom status, functional status, general health perceptions, and overall quality of life (14). (**Figure 2**) Applied to the diabetic hand, most existing scores focus on functional status or pain, assessing how patients manage daily activities or experience discomfort. To our knowledge, no instrument attempts to quantify the biology of the condition, specifically inflammation and collagen deposition, and resultant resistance to stretch. Symptom status and functional status are both influenced by patient-related factors such as the patients’ pain tolerance, symptom amplification, and environmental factors such as social and psychological support. Due to the variation that these influences would bring, they might not be good candidates for measures to correlate with other similar, biological processes.

The validity data increase confidence that we are measuring the construct of interest. In this paper, we show that MCP correlated with a physician’s idea of fibrosis severity, even though this assessment is a far more subjective measure. We consider the correlation of −0.5 to be a reasonable indicator of convergent validity in this context. Given the inherent subjectivity of physician judgment and its ordinal nature, a moderate correlation still supports construct validity—especially when the directionality is consistent and statistically significant. A stronger testament to the validity of this metric comes from our previous work in type 1 diabetes, where mean MCP extension correlated with fibrous tissue deposition on MRI in flexor compartments (9). In addition, weak correlations with both DHI and grip strength denote that these are measuring diverging constructs. Clinically, DHI elevation in those with MCP extension <30° indicate functional impairment only in the most severely affected. This is likely because limited MCP hyperextension alone may not affect most hand functions assessed by DHI, which involve grip and dexterity. In more severe cases, associated flexor contractures of the proximal or distal interphalangeal joints may develop and contribute more directly to functional limitation. Overall, these data suggest that mean MCP extension adds additional value in the clinical armamentarium assessing diabetes hand. While no established minimal clinically important difference (MCID) exists for MCP extension in this context, our findings suggest that a difference of approximately 8°–10° is easily detectable and associated with clinical change over time. In our data, a threshold of <40°for mean MCP extension showed excellent agreement between raters in identifying restricted motion (Cohen’s κ = 0.81) and could serve as a pragmatic cutoff to define “restricted” extension. Future work should validate these thresholds in relation to patient-reported outcomes and functional measures to improve clinical decision-making and screening applicability.

Detecting an ongoing fibrotic process in its preclinical stages is useful because fibrosis, once established, is largely irreversible. Early detection may allow for timely intervention, potentially halting or slowing disease progression before irreversible tissue damage occurs. Using current diagnostic paradigms in the diabetic hand, patients will only be detected when they get the full-fledged manifestation and complain to the physician. Data suggest that end-stage phenotypes, such as the prayer sign, do correlate with stiffness in other tissues. For example, the prayer sign has been associated with difficult endotracheal intubation in surgery (15) and with increased ventilatory hours following bypass surgery (16), possibly suggesting upper respiratory and lung stiffness respectively. The prayer sign is a subjective binary sign for flexor compartment fibrosis, and such associations may benefit from quantitative measures of the same. Having a quantitative measure is also likely to help in evaluating the longitudinal course of these syndromes, which is not possible with binary outcomes. To this effect, we saw that MCP extension is sensitive to change—both in documenting a gradual decline in those with diabetes-related hand conditions and an improvement in more acute flexor tenosynovitis.

Mean MCP extension restriction before symptoms or functional deficits arise could potentially help in detecting such “preclinical” disease. We used the additional category of “skin thickening” as a possible preclinical state before the prayer sign is evident. Mean MCP extension was restricted in this state as compared with controls. It was also interesting to note that the group with prediabetes had a higher prevalence of ST whereas the fraction of LJM increased in type 1 and type 2 diabetes. This observation may suggest that the fibrotic program in the hand is set before hyperglycemia develops, which then modifies it toward increasing the severity of LJM. It will be informative to follow the group with prediabetes and skin thickening, to confirm this possibility. These data will also support a Mendelian Randomization in the UK Biobank, which showed a causal relationship between hyperglycemia and hand manifestations (17).

Reliability studies across a heterogeneous patient population increase our confidence in being able to use mean MCP extension in the clinic and community. While interrater agreement was good overall, the ability in identifying severe extension (<40°) was excellent. With proper training, mean MCP extension can potentially be measured by paramedical staff in the community.

This paper has several strengths, including a large, prospective dataset of more than 2,500 individuals, and the use of blinded clinical opinions to minimize bias. The inclusion of both types of diabetes, prediabetes, and healthy controls enhances generalizability, and the use of smaller datasets tailored to the methodological approach helps establish reliability and responsiveness. The study’s weaknesses include small numbers of patients with certain hand conditions like CTS, the absence of a gold standard imaging technique, lack of correlation with similar scores like the Hand Mobility in Scleroderma (HAMIS) score (18), and the absence of long-term follow-up in most of the cohort. We acknowledge that goniometric measurements are the established standard for assessing joint range of motion. A formal comparison with standard goniometry or digital angle measurement tools would enhance external validity and should be pursued in future studies. Nevertheless, the high inter-rater reliability observed in our study supports the reproducibility of the method with minimal training. Mean MCP extension is not specific to diabetes-related hand conditions. It may be affected by other disorders such as rheumatoid arthritis, neurological contractures, trauma, or palmar psoriasis. As such, its use should be limited to well-characterized populations unless supported by appropriate differential diagnosis. Caution should be exercised when generalizing its application beyond diabetes-related fibrosis without further validation.

Expanding the dataset to include broader hand conditions will help establish ranges.

A further limitation is the absence of imaging and systemic biomarker correlation in the present dataset. While these data would strengthen mechanistic interpretation, the aim of this study was primarily methodological, to establish validity, reliability, and responsiveness of mean MCP extension using COSMIN guidance in a large and diverse cohort. Imaging-based validation is inherently feasible only in smaller subsets. Future work will incorporate magnetic resonance imaging (MRI) as a gold standard that will add to the construct validity, as well as compare the measurement method with goniometry. Long-term follow-up studies in diabetic cheirarthropathy are few and are needed to assess the utility of detecting “preclinical” hand stiffness. Finally, it would be important to correlate mean MCP extension with markers of internal organ fibrosis including the heart, kidney, lung, and liver, to evaluate if the hand can indeed serve as a clinical biomarker (7).

In conclusion, this group of studies highlights the clinical significance of mean MCP extension as a valid indicator of flexor fibro-inflammatory hand manifestations in diabetes, despite their clinical heterogeneity. A notable 20° difference in those with hand manifestations can be easily detected in the community. The measurement can be used reliably with minimal training and is sensitive to both worsening and improvement in these conditions. The large dataset, blinded assessments, and inclusion of controls strengthen the findings and establish a proof of concept for more widespread use of this simple measure in further correlative and interventional studies.

## Data Availability

The raw data supporting the conclusions of this article will be made available by the authors, without undue reservation.
